# Identification and characterization of microglia/macrophages in the granuloma microenvironment of encephalic schistosomiasis japonicum

**DOI:** 10.1186/s12879-019-4725-5

**Published:** 2019-12-30

**Authors:** Zhoubin Tan, Zhuowei Lei, Zhuo Zhang, Huaqiu Zhang, Kai Shu, Feng Hu, Ting Lei

**Affiliations:** 1 Sino-German Neuro-Oncology Molecular Laboratory, Department of Neurosurgery, Tongji Hospital, Tongji Medical College, Huazhong University of Science and Technology, Wuhan, China; 2 Department of Orthopedics, Tongji Hospital, Tongji Medical College, Huazhong University of Science and Technology, Wuhan, China; 3 Present Address: Department of Neurosurgery, Philipps University Marburg, Baldingerstr, Marburg, Germany

**Keywords:** Encephalic schistosomiasis japonicum, Granuloma microenvironment, Microglia/macrophages, Polarization

## Abstract

**Background:**

Egg-induced immune response and granuloma formation are thought to be the basis of central nervous system (CNS)-related clinical symptoms of *Schistosoma japonicum*. Microglia/macrophages are the major immune cells involved in detection and subsequent elimination of pathogens and injured tissue in the brain. However, little is known about their role in the pathogenesis of neuroschistosomiasis. The main purpose of the study is to clarify the pathological involvement of microglia/macrophages in the pathogenesis of neuroschistosomiasis (NS).

**Methods:**

Staining techniques were applied to the granuloma tissues excised from 4 patients, as well as mice model which was established by microinjecting viable *S. japonicum* eggs into the brain. Clinical features of the patients and neurological symptoms in mice were also collected and analyzed in terms of their correlation with microglia/macrophages.

**Results:**

Microglia/macrophages constituted the major portions of the granulomas surrounding the eggs in both all human cases and *S. japonicum* egg-injected mice. Granuloma persisted in all patients accompanied by unremitted neurological symptoms, while in mice granuloma formation initiated on day 3, peaked on day 7 and subsided on day 30 post injection with *S. japonicum* eggs. No neurological abnormalities were observed in egg-injected mice except for significant weight decrease on day 3 compared with saline-injected control. M1 polarization of microglia/macrophages was confirmed in egg-injected mice 3 days post injection and in all human cases. M2 polarization was absent in human patients despite the duration of complaints but dominated in the whole progression of egg-induced pathology in mice until the elimination of eggs and subsidence of neuroinflammation on day 30 post injection.

**Conclusions:**

Microglia/macrophages participated actively in the granuloma microenvironment of encephalic schistosomiasis japonicum in both human and mice. The polarization pattern of microglia/macrophages coincided with the symptomatic features in human cases and *S. japonicum* egg-injected mice, indicating M2 instead of M1 activation as a probably more important mediator in the battle against egg-induced pathology and concomitant manifestations. These new findings will shed light on the pathogenesis of NS from a brand-new perspective, and may contribute to the immunotherapy development for such disease, favoring perhaps M2 polarization of microglia/macrophages as a feasible strategy.

## Background

Schistosomiasis is a prevalent parasitic disease which plagues more than 240 million people in 76 countries in the world [1]. Neuroschistosomiasis (NS), referring to schistosomal involvement of the CNS (central nervous system), is a severe form of presentation of schistosomiasis [2]. *Schistosoma japonicum* (*S. japonicum*), prevalent in Southeast Asia, mainly affects the brain (encephalic schistosomiasis japonicum), which constitutes 2–4% of all such cases [3, 4]. Although the pathogenesis of NS has not been fully elucidated, it is proposed that the deposition of parasite eggs in the nervous tissue induces an inflammatory response leading to the formation of granulomas, which is the basis of CNS-related clinical symptoms [2–5]. Common manifestations in CNS-infected patients include headache, vomiting, dizziness, convulsion and paralysis, adding to the global burden of schistosomiasis [6]. Despite the great progress in controlling the transmission of *Schistosoma japonicum* in China, there remains little known about the basic mechanisms underlying the pathophysiology of CNS infection [7].

Microglia/macrophages are the major immune cells involved in detection and subsequent elimination of pathogens and injured tissue in CNS [8]. However, very little is known about their roles in the granuloma formation surrounding the eggs and clinical significance in CNS. In order to clarify the pathological involvement of microglia/macrophages in the pathogenesis of NS, as well as microglia/macrophages correlation with clinical features, staining techniques were applied to the granuloma tissues excised from 4 patients, obtained during neurosurgical operation in present study.

There are animal models of schistosomiasis presenting CNS infection. However, CNS involvement was very rare in natural infection progression, which contributed to the difficulty of research on it [9–11]. Microinjection of viable *Schistosoma* eggs into specific organs to establish schistosomiasis animal models has been reported to be a useful way in studying site-specific infections of *Schistosoma* [12–15]. Herein we apply microinjection technique in establishing encephalic schistosomiasis japonicum mice model to investigate the role of microglia/macrophages in its pathogenesis.

## Methods

### Clinical data

The study was conducted on tissues obtained from 4 cases of encephalic schistosomiasis japonicum. Associated clinical data was collected from archival files dating from 2013 to 2017 in the Department of Neurosurgery, Tongji Hospital, Tongji Medical College, Huazhong University of Science and Technology. The clinical features of these cases are summarized in Table 1.

**Table 1:** Summary of clinical characteristics of encephalic schistosomiasis japonicum

Case No.	Sex	Presenting symptoms	Site	Therapy performed	Follow-up
1	M	Headache with vomiting for 9 days	Single nodule in right cerebellum	Total surgical resection	Well-controlled 4 years with continuous follow-up
2	F	Epilepsy for 3 days	Single nodule in left parietal lobe	Total surgical resection	Seizure-free 2 years, loss of contact afterwards
3	M	Headache and motor aphasia for 20 days	Multiple nodules in left frontotemporal lobe	Total surgical resection	Loss of contact
4	M	Epilepsy for 1 week	Multiple nodules in left frontal, parietal and temporal lobes	Total surgical resection	Seizure-free 2 years with continuous follow-up

### *S. japonicum* egg isolation

*S. japonicum*-infected rabbits were generously provided by Dr. Fei Guan and Dr. Junli Xiao at the Department of Parasitology, Tongji Medical College, Huazhong University of Science and Technology. The rabbits were subcutaneously sedated with a ketamine/xylazine mix solution (50 mg/kg and 3 mg/kg, respectively) followed by intra-cardiac injection of sodium pentobarbital 6 weeks post-infection. The livers were minced, homogenized in a blender, resuspended with 1.2% NaCl solution containing antibiotic-antimycotic solution (100 units Penicillin, 100 μg/mL Streptomycin and 0.25 μg/mL Amphotericin B, Sigma-Aldrich) and 0.25% trypsin, passed through a series of sieves with sequentially decreasing pore sizes (500 μm, 200 μm, and 100 μm), and finally retained on a 45 μm sieve. The eggs were repeatedly washed to remove impurities and resuspended in 4 °C 0.9% NaCl solution for injection. Control group was applied with equal volume of 0.9% NaCl.

### Mice model establishment

Six to seven week-old female C57BL/6 mice were purchased from Animal Center of Tongji Hospital. Forty mice were randomly assigned into Egg and control groups. Animals were anesthetized with isoflurane and placed in a stereotactic frame. The skin was incised and one hole was drilled in the skull. Freshedly prepared *S. japonicum* eggs (500 eggs in 2 μl of 0.9% NaCl) or saline was injected into the cortex. Each injection lasted 4 min with the needle left in situ for another 2 min, then raised 0.5 mm and left another minute, before being slowly withdrawn. The incisions were closed with suture and treated once with topical antibiotic ointment. Neurological symptoms and weight was closely recorded after surgery. Mice were euthanized by CO_2_ at serial time points 3 to 30 days after brain injection, and brains processed for further histological analysis. All experimental procedures were carried out in accordance with the Institutional Animal Care and Use Committee guidelines and approved by Ethical Committee of Tongji Hospital, Tongji Medical College, Huazhong University of Science and Technlogy. Full exertions were made to lessen animal suffering.

### Histopathology, immunohistochemistry and immunofluorescence

The tissues were routinely fixed in 10% buffered formalin for at least 12 h, and processed and embedded in paraffin blocks; 5 μm paraffin sections were microtomed, gradually dewaxed into the water, and HE staining was performed. The histopathological features were evaluated under light microscope (Nikon 80i).

For immunohistochemistry, the paraffin sections were gradually dewaxed into the water, then immersed in 0.01 mol/L citrate buffers and microwaved for antigen retrieval. After incubation of primary antibodies on the sections according to the protocol from the suppliers, the standard DAKO ChemMate™ EnVision Kit (horseradish peroxidase (HRP)/3,3′-diaminobenzidine (DAB), rabbit/mouse) (Shanghai Gene Company, Shanghai, China), based on the two-step labeled HRP method, were used according to the manufacturer’s instructions. PBS solution instead of the primary antibody was used as the negative control during immunohistochemical staining. DAB was used as the chromonogen. The deposition of granular dark brown pigments were considered as positive staining.

For immunofluorescence, the paraffin sections were gradually dewaxed into the water, then immersed in 0.01 mol/L citrate buffers and microwaved for antigen retrieval. After incubation of primary antibodies on the sections according to the protocol from the suppliers, the secondary antibodies labelled with specific fluorescent compounds were used. PBS solution instead of the primary antibody was used as the negative control. DAPI was used as the chromonogen. Images were captured using fluorescence microscope (Leica AF 6000).

The primary antibodies used in this study are summarized in Table 2.

**Table 2:** Primary antibodies used in this study

Primary antibody	Dilution	Company
IBA1 ab5076	1:500	Abcam
iNOS ab178945	1:500	Abcam
Arg1 ab212522	1:500	Abcam

### Statistical analysis

Unpaired t test with Welch’s correction was used to compare the results between control and egg-injected groups, and data were expressed as mean ± standard deviation. P<0.05 was considered statistically significant.

## Results

### Clinical features

The age of the patients in this group ranged from 27 to 51 years (median age of 47.25 years). The male to female ratio was 3:1. Every patient had a history of contact with schistosome-infected water. None of the patients presented with gastrointestinal manifestations such as hepatosplenomegaly, ascites and esophagogastric varices. In contrast, all of the patients presented with headache and two of them presented with seizures, with one presenting with motor aphasia. The duration of their complaints ranged from 3 to 20 days. All the patients received anti-schistosomal medical treatment but remission was not achieved and thus surgery was performed.

### Preoperative neuroradiology

**Fig. 1:**
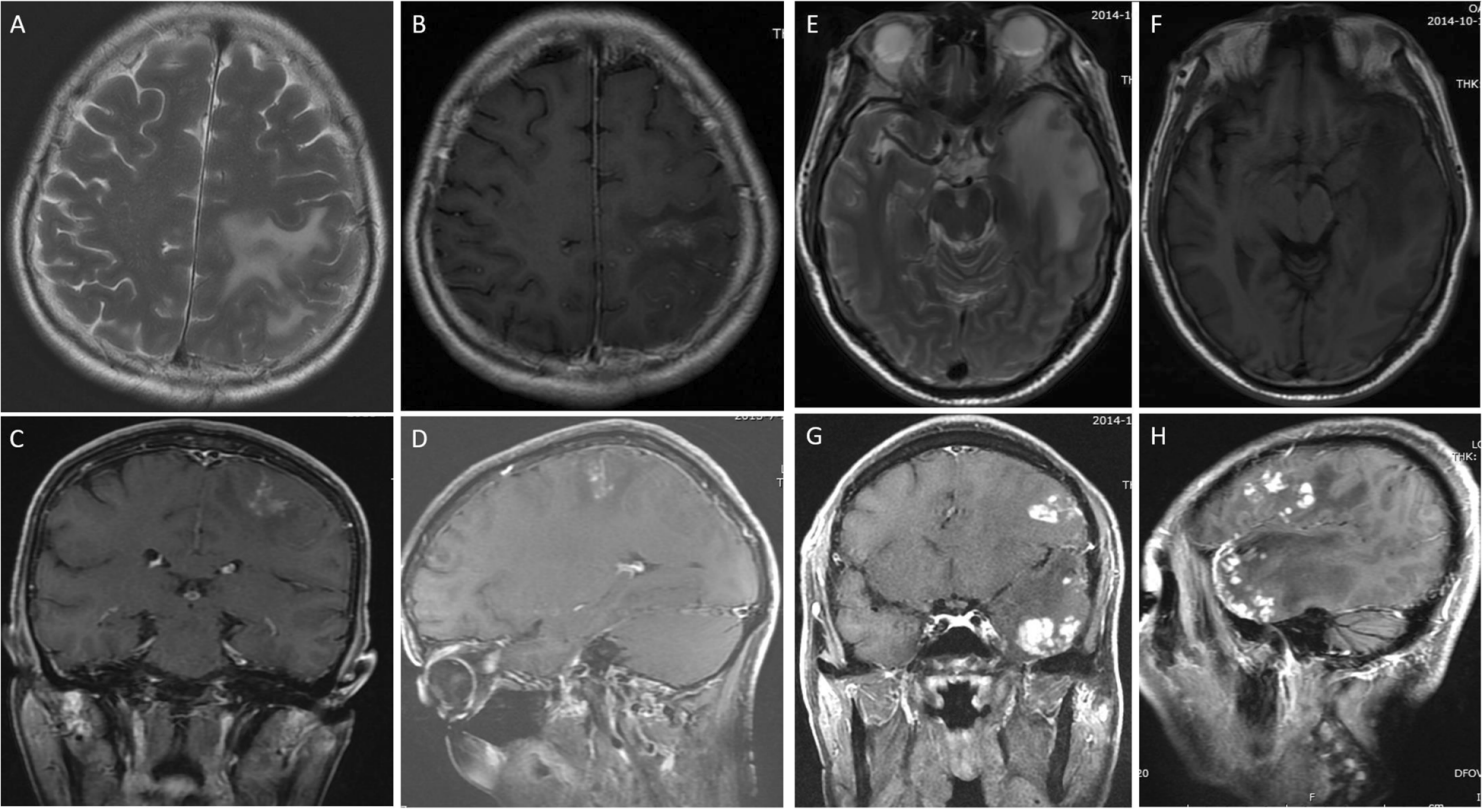
MRI features of the representative encephalic Schistosomiasis japonicum cases (**a-d** from Case 2, **e-h** from Case 4). **a**: T2 weighted (W) image showed a single-nodular lesion located in the left parietal lobe with perilesional edema by axial scanning. **b-d**: T1 W image after gadolinium injection showed single-nodular enhancement with perilesional edema by axial (**b**), coronal (**c**), and sagittal scanning (**d**). **e**: T2 W image showed edema of left temporal lobe by axial scanning. **f**: T1 W image showed the involvement of the left temporal lobe in multi-nodular lesions by axial scanning. **g, h**: T1 W image showed multiple-nodular schistosomal lesions located in frontal, parietal and temporal lobes with enhancement of the nodules

MRI scans of the patients revealed either single or multiple nodular schistosomal granulomas (Fig. 1). Depending upon the patient, the nodules were located uniquely or in combination (multiple) in one side of the parietal, frontal and/or temporal lobes and/or cerebellum. For example, in case 2 (Fig. 1a-d), the lesions were located only in the left parietal lobe. In contrast, in case 4 (Fig. 1e-h), multiple lesions were found in the left frontal, parietal and temporal lobes. T2WI (T2-weighted image) showed edema of differing extents surrounding the nodules. Enhanced scanning indicated enhancement of the nodules.

### Pathological features of brain tissue from neuroschistosomiasis patients

**Fig. 2:**
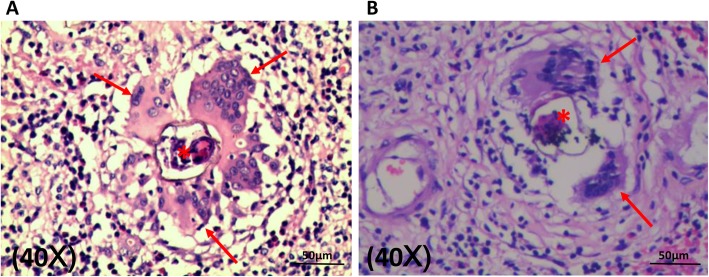
Pathological features of brain tissue from neuroschistosomiasis patients (**a** from Case 1, **b** from Case 2). Schistosomal eggs were surrounded by multinucleated giant cells. Asterisk = *S. japonicum* egg, Arrow = multinucleated giant cells

Microscopically, presence of parasite eggs was clearly observed in all cases (Fig. 2). Histological staining demonstrated that the observable eggs were surrounded by multinucleated giant cells indicating an immune response forming granulomas.

### Assessment of microglia /macrophage abundance in the brain tissue from neuroschistosomiasis patients

**Fig. 3:**
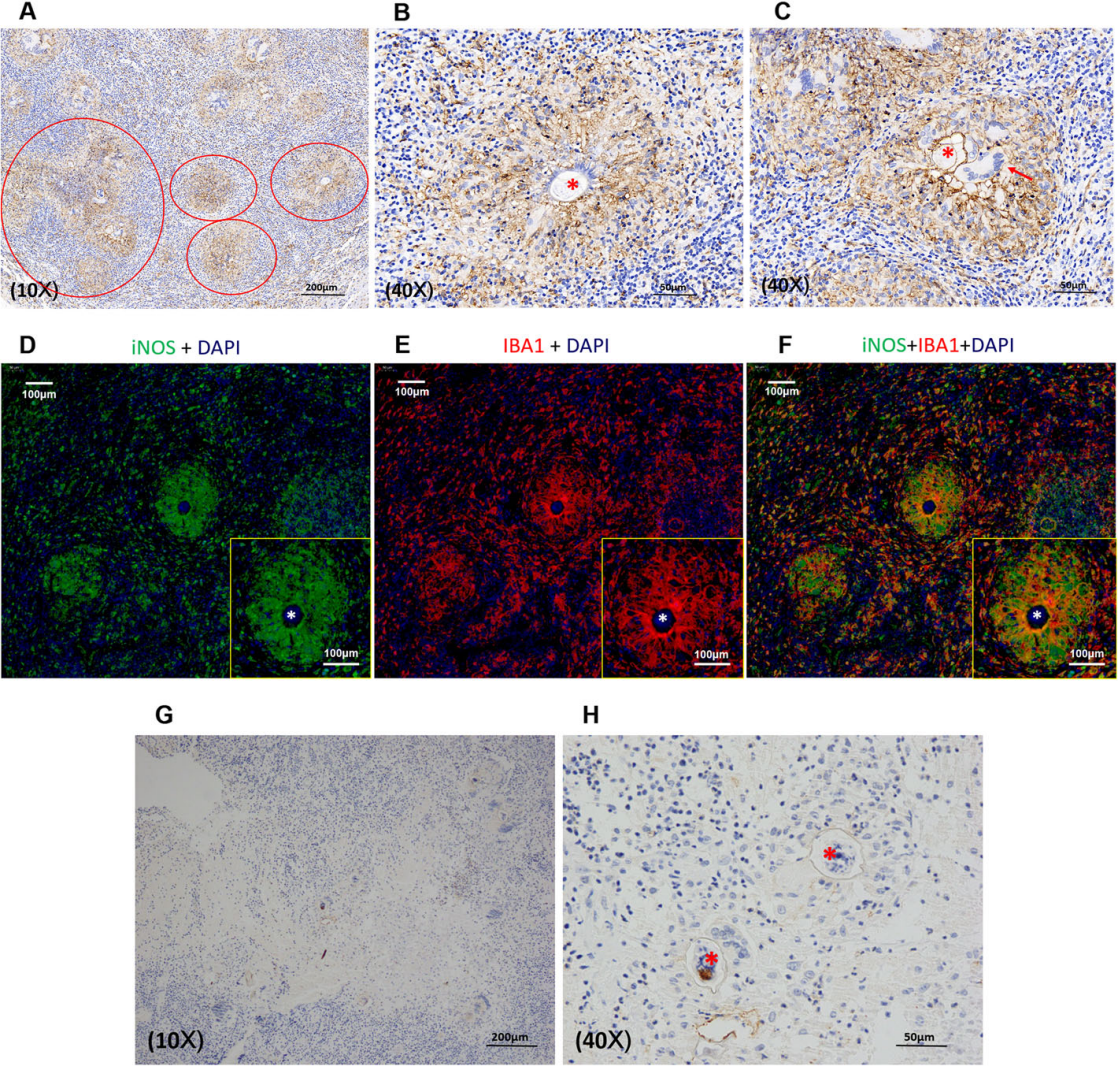
Identification and polarization of microglia/macrophages in the granuloma microenvironment of neuroschistosomiasis patients. **a-c**: Microglia/macrophages constituted the major portions of the granulomas (*circle*) surrounding the eggs as highlighted by IBA1 staining. The schistosomal egg shell (*asterisk*) can be observed clearly with multinucleated giant cells (*arrow*) in the vicinity. **d-f**: Immunofluorescent staining indicating M1 polarization of microglia/macrophages. iNOS (green) and IBA1 (red) were well co-expressed in the vicinity of the eggs (*asterisk*). Merged image demonstrated that the same cells were exactly the M1 type microglia/macrophages. **g-h**: Immunohistochemical staining illustrating negative expression of M2 marker (Arg1) of microglia/macrophages surrounding the eggs (*asterisk*)

In all 4 cases, the granulomas appeared to be accumulated by microglia/macrophages surrounding the eggs as indicated by large amounts of dark brown staining using an antibody against the specific IBA1 (Ionized calcium binding adapter molecule 1) marker [16] for such cells (Fig. 3a-c). Absorption control staining was carried out to further confirm the specificity of the antibody (Additional file 1: Figure S1).

### Polarization of microglia/macrophages in CNS Schistosomiasis patients

The polarization of the microglia/macrophages was determined using specific antibodies for M1 marker (iNOS, inducible nitric oxide synthase) and M2 marker (Arginase1, Arg1) and merging the images with that given by IBA-1 (Fig. 3d-h). Interestingly, in all cases the polarization of the microglia/macrophages was of the M1 type, the staining being negative for Arg1. The merged image demonstrated that the same cells were positively stained for both iNOS and IBA-1 (Fig. 3d-f).

### Neurological abnormalities of mice model

**Fig. 4:**
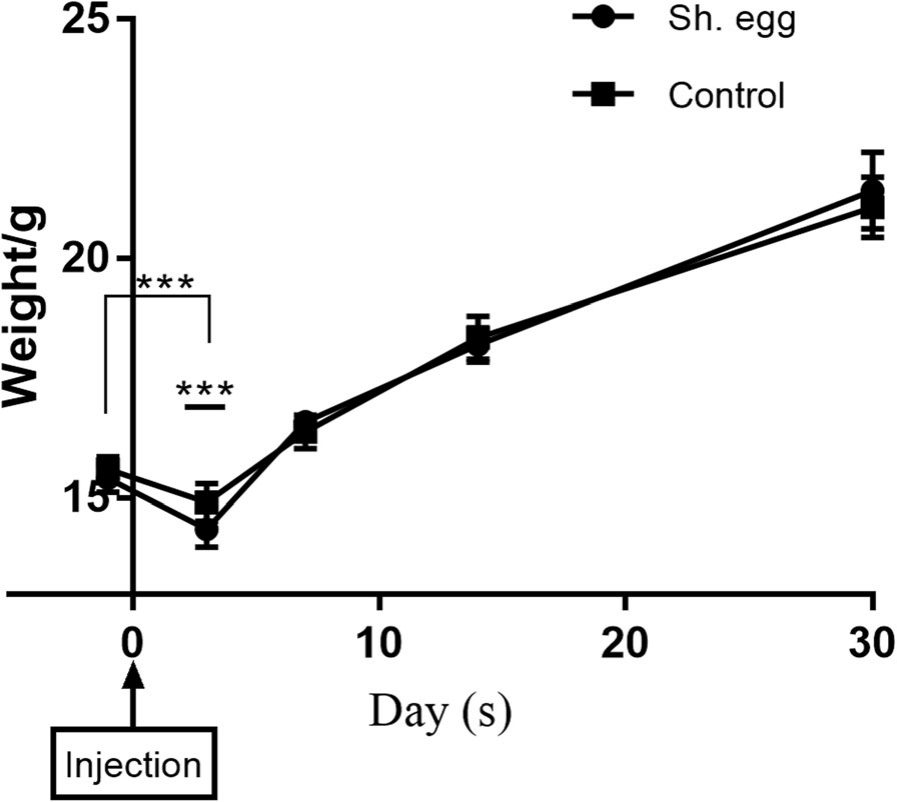
Weight of egg-injected and control groups over time. Unpaired t test with Welch’s correction was used to compare the weight between the two groups, and data were expressed as mean ± standard deviation. P<0.05 was considered statistically significant. The data showed significant decrease of the weight on day 3 in both groups compared to day 0 (*** *P* < 0.001) and significant difference in the weight of two groups on day 3(*** P < 0.001)

Although no neurological symptoms like seizure and hemiplegia were observed in either of the groups after recovery from anesthesia, the weight of both groups decreased significantly in 3 days post-operation (P<0.001) (Fig. 4), and increased steadily after that. More interestingly, there was significant difference in the weight of two groups 3 days post-operation with egg group decreasing in a greater degree (P<0.001) (Fig. 4).

### *S. japonicum* egg injection-induced pathological process in mice

**Fig. 5:**
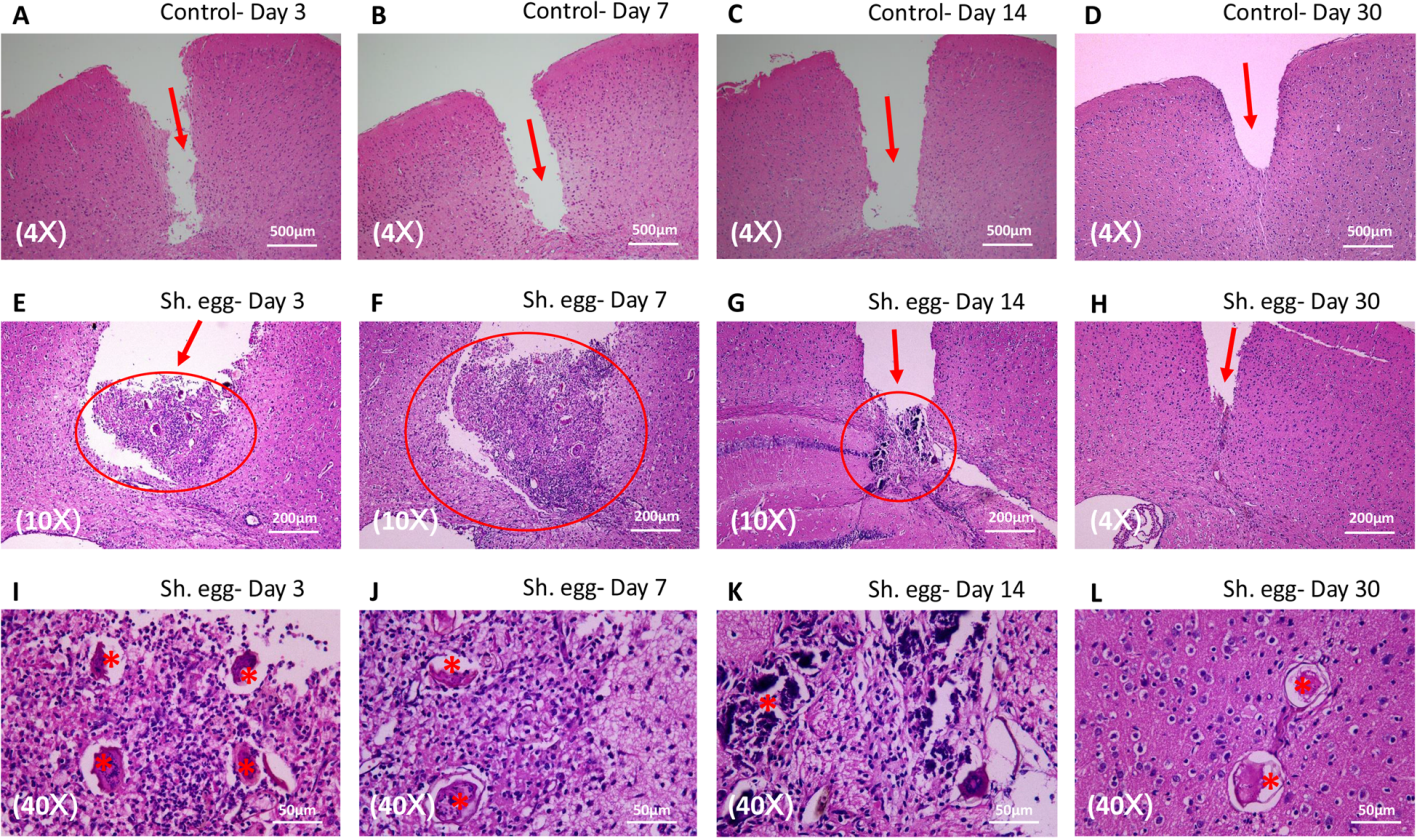
*S. japonicum* egg injection-induced pathological process in mice. (**a-d**): Injection site response resolved in egg-free control group, leaving puncture path visible (*arrow*). Granuloma formation (*circle*) initiated on day 3, peaked on day 7 and subsided after day 14 (**e-h**). Inflammatory infiltrate can be observed surrounding the eggs (*asterisk*) (**i-k**). Egg disintegration occurred on day 14 (**g, k**) and elimination on day 30 (**h**). Unviable *S. japonicum* eggs (*asterisk*) without inducing immune response can be found on day 30 (**l**)

As evidenced by results from encephalic schistosomiasis japonicum patients, pathogenesis and concomitant morbidity is the result of *S. japonicum* egg deposition in cerebral tissue. However, the whole pathological process of cerebral infection has never been reported and thus hindered its further investigation. To address this issue, we directly microinjected freshly-prepared viable *S. japonicum* eggs into the cerebral cortex of female C57BL/6 mice. The initial injection site response resolved entirely by day 3 in mice injected with egg-free control vehicle, leaving puncture path visible (Fig. 5a-d). Egg-injected group demonstrated inflammatory infiltrate and granuloma formation surrounding *S. japonicum* eggs as early as day 3 (Fig. 5e and i). Inflammation response and granuloma continued to progress and peaked on day 7 (Fig. 5f and j). Interestingly, unlike findings in humans, *S. japonicum* egg disintegration was observed on day 14 and inflammation response diminished greatly (Fig. 5g and k). On day 30 the inflammation subsided completely and *S. japonicum* eggs vanished without trace (Fig. 5h) except for the ones which we think were unviable and unable to secrete soluble antigen to induce immune response (Fig. 5l).

### Microglia /macrophage accumulation in mice model

**Fig. 6:**
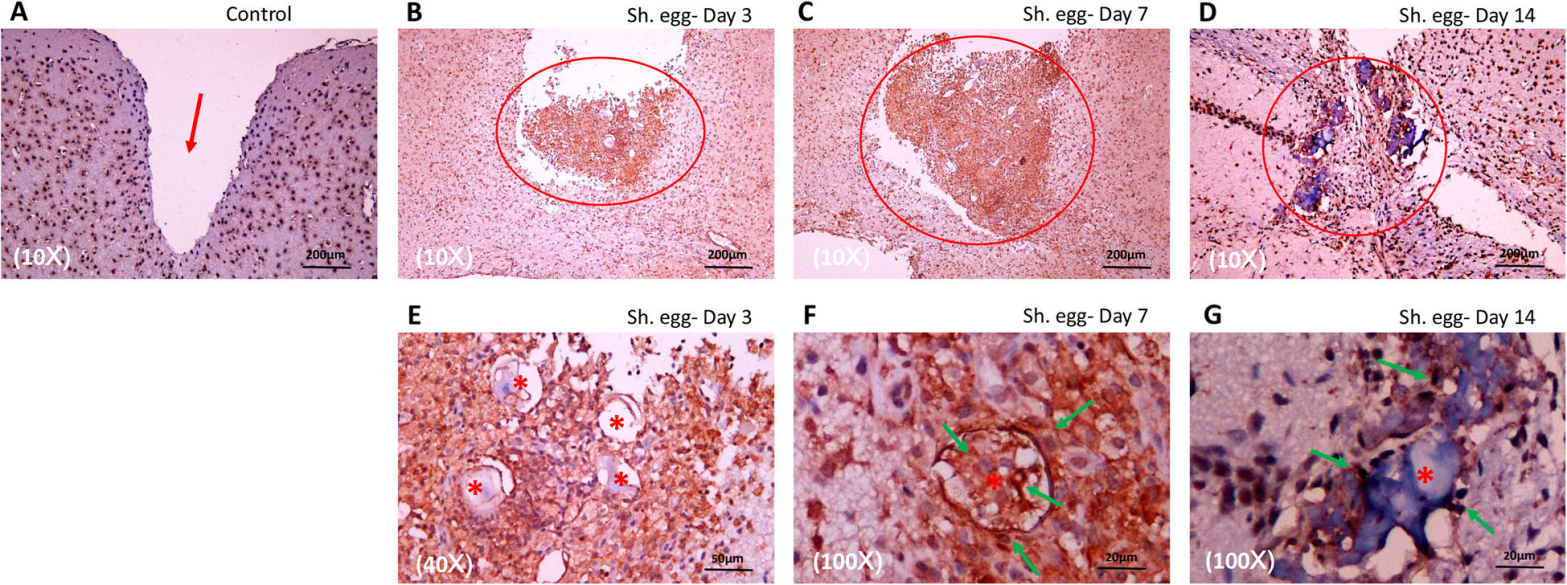
Immunohistochemical staining illustrating active participation of microglia/macrophages in egg-injected mice model. No microglia/macrophages-formulated granuloma was detected in control (**a**). Granuloma (*circle*) was mostly composed of microglia/macrophages in egg-injected mice as indicated by specific IBA1 marker (**b-d**). Microglia/macrophages (*arrow*) acted as the first line immune cells in the battle against *S. japonicum* eggs (**e-g**)

In accordance with results in encephalic schistosomiasis japonicum patients, granulomas appeared to be mostly composed of microglia/macrophages surrounding the eggs as confirmed by immunohistochemistry for IBA1 on day 3 and day 7 (Fig. 6bc), while control exhibited neither granuloma formation nor microglia/macrophages aggregation (Fig. 6a). With the decomposition of *S. japonicum* eggs on day 14, granuloma shrank and microglia/macrophages abundance decreased significantly (Fig. 6d). *S. japonicum* eggs were always closely surrounded by microglia/macrophages (Fig. 6e-g). During egg elimination process, microglia/macrophages could even invade inside the eggs (Fig. 6f). These findings added to the fact that microglia/macrophages participated actively in the *S. japonicum* egg-induced immune response and played a primary role in the elimination of *S. japonicum* eggs.

### Polarization of microglia/macrophages in *S. japonicum* egg-induced granulomas in mice

**Fig. 7:**
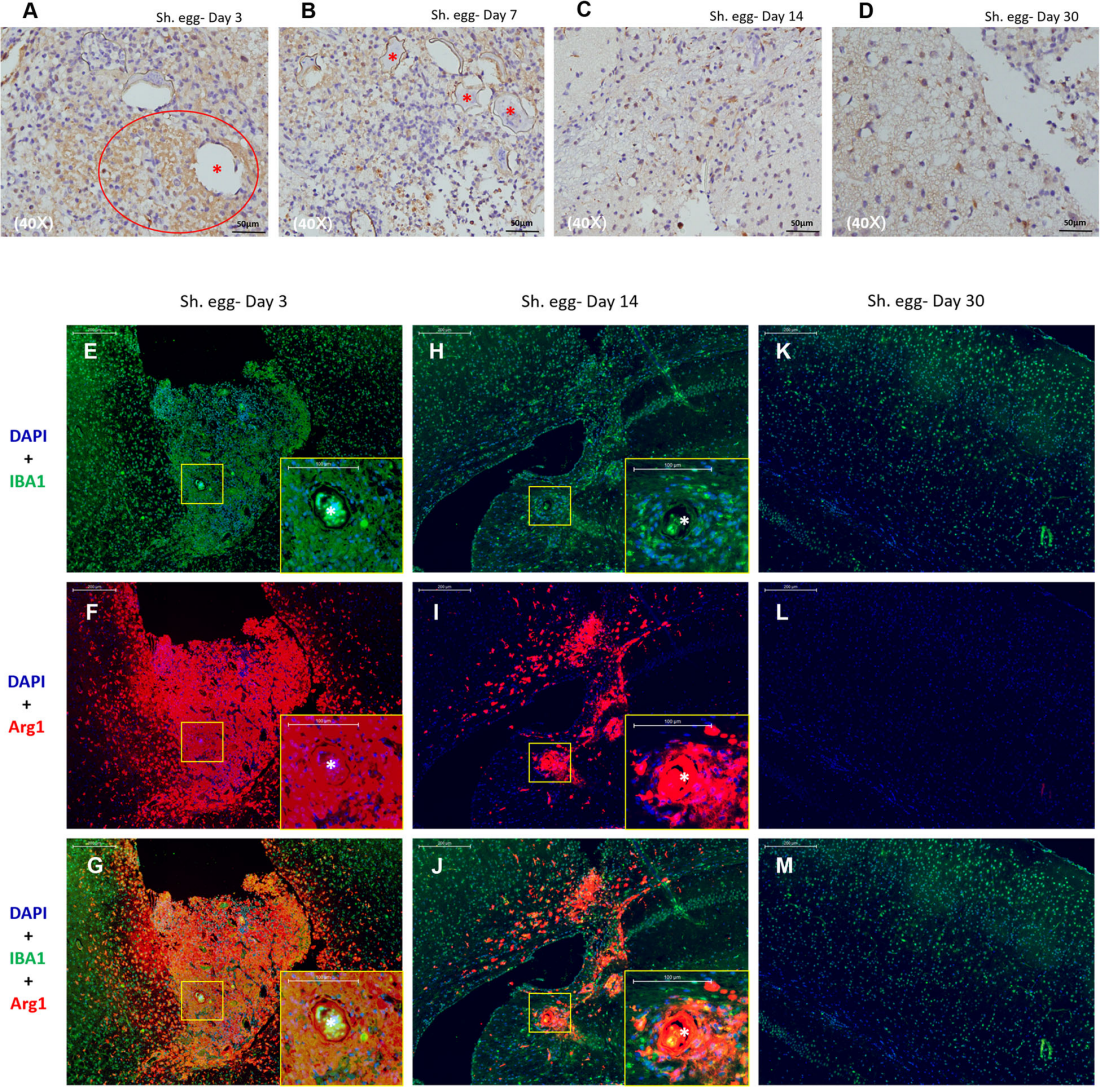
Polarization of microglia/macrophages in *S. japonicum* egg-induced granulomas in mice. **a-d**: Immunohistochemical staining illustrating M1 polarization of microglia/macrophages on day 3 in egg-injected mice as indicated by positive iNOS expression (*circle*) surrounding the eggs (*asterisk*). **e-m**: Immunofluorescent staining indicating M2 polarization of microglia/macrophages in egg-injected mice. M2 polarization sustained from day 3 until inflammation subsidence on day 30, as indicated by positive expression of both Arg1 (*red*) and IBA1 (*green*). *S. japonicum* eggs: Asterisk

M1 polarization of microglia/macrophages existed in *S. japonicum* egg-induced granulomas only on day 3 post injection (Fig. 7a) and specific marker iNOS staining was negative after that (Fig. 7b-d), while M2 polarization sustained from day 3 until inflammation subsidence on day 30, as indicated by positive expression of both Arg1 (red) and IBA1 (green), colocalization of which confirmed M2 microglia/macrophages in the vicinity of the eggs (Fig. 7e-m).

## Discussion

Schistosomiasis japonicum is an important disease to investigate since it is of prevalence in Asian countries and can infect workers such as rice farmers and fishermen, who are an integral part of the food economy in these countries [17]. The involvement of the CNS, which is relatively rare, can cause a series of the most harzardous symptoms such as epilepsy, hemiplegia and intracranial hypertension contributing to the medical burden [3, 4]. It is surprising, therefore, that there is a paucity of clinical and scientific investigations of this disease in the literature, although the life cycle of the parasite is quite well established [17]. It is hypothesized by some researchers that the eggs can invade the CNS from the portal mesenteric and pelvic system through two routes: Batson’s venous plexus and the arterial system [2, 18]. The trapped eggs can induce a host’s immune response and cause the formation of granulomas whose mass effect and intrinsic immune properties are thought to be the basis of clinical symptoms [2–4, 18, 19]. Although there is a lack of study thoroughly demonstrating the microenvironment of the granuloma formation in encephalic Schistosomiasis japonicum, researches focusing on Schistosomiasis mansoni (another species of genus *Schistosoma*, mainly prevalent in Africa and Brazil) revealed the involvement of monocytes, lymphocytes and eosinophils along with soluble factors in CNS infection [5]. Microglia/macrophages are among the first effectors to pathogen invasion, responding via releasing of various cytokines and clearance of pathogens and debris [20, 21]. However, none of the investigation mentioned the role of microglia/macrophages in the granuloma microenvironment in neuroschistosomiasis. Therefore, we undertook this present study in order to gain a better understanding of the disease process with the emphasis on the role of microglia/macrophages in such disease. The findings demonstrate that the lesions can occur either at single or multiple sites in the brain and lead to typical immune type histological changes in patients, where multinucleated giant cells, which is thought to be the result of fusion of microglia/macrophages, [22] can be found to be located closely to the eggs. In addition, our series of patients as well as mice model showed, for the first time, that microglia/macrophages constituted the major portions of the granulomas surrounding the eggs, demonstrating their important role in this disease. In egg-injected mice, microglia/macrophages could invade inside the eggs on day 7 post injection, followed by the decomposition and elimination of eggs on day 14, revealing their active participation in mediating local pathogen control.

During the course of infection, the symptomatic and pathological features of mice model were different from those of CNS-infected patients in many aspects. The inflammation and granuloma formation in mice model existed in early stage (< 14d), which resembled findings in patients as revealed by both neuroradiology and microscopy, and subsided completely on day 30. However, inflammation and granuloma persisted without signs of remission even when anti-schistosomal medical treatment was applied in all the patients. More interestingly, the injected eggs gradually lost their original shape over the course, disintegrated and disappeared in the end, while in all human cases eggs persisted despite the duration of their complaints and anti-schistosomal medical treatment. Moreover, no neurological symptom was observed in mice model in contrast to 4 cases who exhibited severe complications such as headache and seizure before neurosurgery. In parallel, reasonably, the weight decrease of control mice on day 3 might be attributed to surgical procedure and confounding factors, while the weight difference between control and egg group at the same time might be due to egg-induced immune response.

Given the importance of microglia/macrophages in the granuloma microenvironment of encephalic schistosomiasis japonicum in both human and mice as evidenced above, we further studied their polarization pattern in attempt to explain the discordance between patients and mice model.

As an important fighter against pathogenic infections in CNS, resting microglia may undertake two activation pathways: the classical known as M1, or the alternative known as M2 [23, 24]. Macrophages, peripheral counterparts of microglia, can infiltrate the brain and contribute to the homeostasis of it together with resident microglia, both of which share similar biological functions and polarization patterns [25, 26]. M1 polarization of microglia/macrophages is thought to be involved in the initiation and perpetuation of neuroinflammation, whereas M2 reaction is more likely related to the remission of neuroinflammation through elimination of pathogens and damaged tissues [8]. The nitric oxide, produced by iNOS (an M1 marker), plays a key role in inhibiting the growth of *Schistosoma japonicum* in rats [27]. It is also well known that M1 microglia/macrophage-related neuroinflammation can lead to secondary brain defects after some conditions such as stroke and traumatic brain injury [28, 29]. M1 phenotype may also create an unfavorable microenvironment for CNS recovery by secreting destructive factors that aggravate neurological injury [30–32]. By contrast, M2 microglia/macrophages can not only resolve neuroinflammation by clearing cellular debris and pathogens but can also promote restorative processes by releasing numerous protective and trophic factors [33–35]. Arginase1, among the first discovered M2 markers, can down-regulate nitric oxide synthesis and initiate tissue regeneration and fibrosis [36]. All the human cases in our present study exhibited a microglial/macrophage mediated response perhaps involving only an M1 polarization (indicated by iNOS) at the time after resection, while in egg-injected mice M1 phenotype existed on day 3 and M2 activation (indicated by arginase1) persisted throughout the immune response until neuroinflammaton subsided on day 30. These findings in combination with the manifestations in human cases and mice model demonstrated a strong possibility that it was the M1 polarization of microglia/macrophage that mediated the persistent neuroinflammation, which further harmed the brain and induced a series of additional symptoms in human cases and weight decrease in egg-injected mice model. M2 activation, unobserved in human patients while dominating in egg-injected mice model, might be the dictator of *S. japonicum* elimination, neuroinflammation remission and absence of symptoms in mice. However, due to the limitation of human samples, all the patients have been treated with different medications such as anti-schistosomiasis drug, steroid or mannitol which may affect the phenotype of microglia in the brain. More patient samples are needed for further identifying the phenotype of microglia/macrophages at different stages of neurochistosomiasis.

The mechanism of microglia/macrophage polarization in CNS-infected schistosomiasis has never been reported. Soluble egg antigen secreted by viable *S. japonicum* eggs can activate M2 macrophages in mice via the STAT6 and PI3K Pathways in vitro [37]. Numerous factors existing in the microenvironment of CNS can polarize microglia/macrophage to different phenotypes [38]. IFN-γ, TNF and IL-2 secreted by type 1 T-helper cells can induces M1 polarization while IL-4 secreted by type 2 T-helper cells and regulatory T cells can cause M1 activation [39, 40]. Soluble factors released from injured neurons and extracellular matrix proteins can induce M1 and M2 polarization respectively [29, 41]. Although the mechanism of microglia/macrophage polarization still needs to be unveiled in neuroschistosomiasis, our present study revealed M2 instead of M1 as a neuroinflammation suppressor and symptom controller in encephalic schistosomiasis japonicum, which will shed light on the possible immunotherapy strategies in the control of such disease.

## Conclusions

This report is the first to thoroughly investigate the clinical features and microglia/macrophages features of both patients and mice model with specific CNS-related *Schistosoma japonicum* infection. We first proved the majority part of the granulomas surrounding the eggs to be composed of microglia/macrophages in human and mice model by staining technique. The polarization pattern of microglia/macrophages was then examined and the manifestations of both patients and mice model were recorded at the same time. M1- dominant polarization in patients and M2-dominant activation in egg-injected mice coincided with the complaints in patients and symptomlessness in mice model, revealing that microglia/macrophages acted as double-edged swords in the battle of *S. japonicum* egg-induced pathology. These new findings will shed light on the pathogenesis of NS from a brand-new perspective, and may contribute to the immunotherapy development for such disease, favoring perhaps M2 polarization of microglia/macrophages as a feasible strategy.

## Supplementary information

**Additional file 1: Figure S1.** Absorption control staining using human Iba1 peptide (Abcam, ab23067) confirming the specificity of Iba1 antibody (Abcam, ab5076). The peptide to antibody mixture was made at a working dilution of 10:1 (molar ratio) and pre-incubated overnight at 4 °C. The pre-absorbed antibody was then incubated with tissue excised from neuroschistosomiasis patients in place of the primary antibody alone. Iba1 staining surrounding the eggs (asterisk) was abolished after the antibody was first pre-absorbed with Iba1 peptide (A, B) compared with Fig. 3a-c.

## Data Availability

The datasets used and/or analyzed in the current study were available from the corresponding author on reasonable request.

## Abbreviations

Arg1: Arginase1
CNS: Central nervous system
IBA1: Ionized calcium binding adapter molecule 1
iNOS: Inducible nitric oxide synthase
NS: Neuroschistosomiasis
