# Supplementary figures and images for: Identification and characterization of microglia/macrophages in the granuloma microenvironment of encephalic schistosomiasis japonicum
Source: BMC Infect Dis. 2019 Dec 30;19:1088. doi: 10.1186/s12879-019-4725-5 (PMC6937796; doi:10.1186/s12879-019-4725-5)

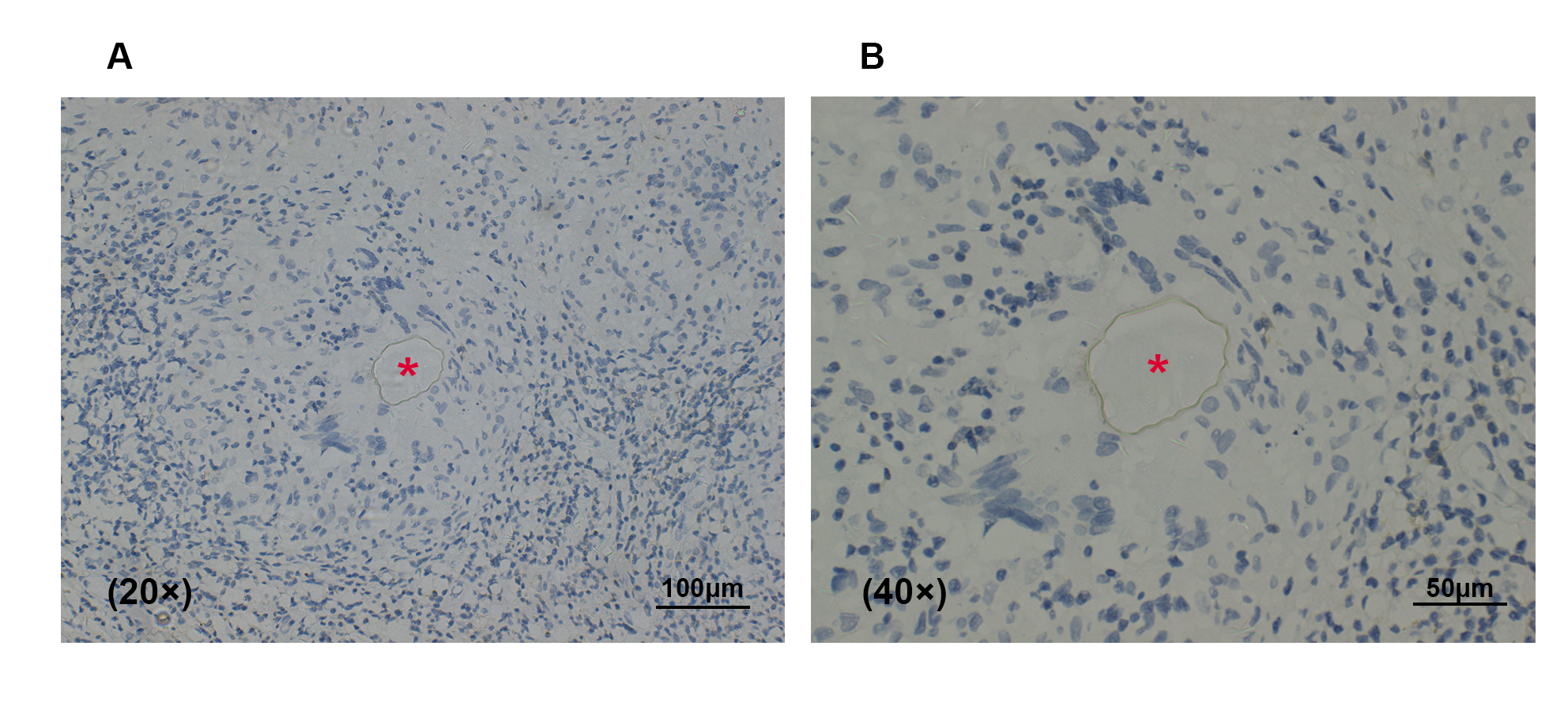

Supplement: Supplementary file 1 — Additional file 1: Figure S1. Absorption control staining using human Iba1 peptide (Abcam, ab23067) confirming the specificity of Iba1 antibody (Abcam, ab5076). The peptide to antibody mixture was made at a working dilution of 10:1 (molar ratio) and pre-incubated overnight at 4 °C. The pre-absorbed antibody was then incubated with tissue excised from neuroschistosomiasis patients in place of the primary antibody alone. Iba1 staining surrounding the eggs (asterisk) was abolished after the antibody was first pre-absorbed with Iba1 peptide (A, B) compared with Fig. 3a-c. [file 12879_2019_4725_MOESM1_ESM.tif]
